# Phylomitogenomic analyses on collembolan higher taxa with enhanced taxon sampling and discussion on method selection

**DOI:** 10.1371/journal.pone.0230827

**Published:** 2020-04-13

**Authors:** Xin Sun, Daoyuan Yu, Zhijing Xie, Jie Dong, Yinhuan Ding, Haifeng Yao, Penelope Greenslade

**Affiliations:** 1 J.F. Blumenbach Institute of Zoology and Anthropology, University of Göttingen, Göttingen, Germany; 2 Key Laboratory of Wetland Ecology and Environment, Northeast Institute of Geography and Agroecology, Chinese Academy of Sciences, Changchun, China; 3 Soil Ecology Lab, College of Resources and Environmental Sciences, Nanjing Agricultural University, Nanjing, China; 4 University of Chinese Academy of Sciences, Beijing, China; 5 Department of Entomology, College of Plant Protection, Nanjing Agricultural University, Nanjing, China; 6 Environmental Management, School of Applied and Biomedical Science, Federation University, Ballarat, Victoria, Australia; 7 Division of Biology, Australian National University, Australian Capital Territory, Australia; Saint Mary's University, CANADA

## Abstract

Collembola are a basal group of Hexapoda renowned for both unique morphological characters and significant ecological roles. However, a robust and plausible phylogenetic relationship between its deeply divergent lineages has yet to be achieved. We carried out a mitophylogenomic study based on a so far the most comprehensive mitochondrial genome dataset. Our data matrix contained mitogenomes of 31 species from almost all major families of all four orders, with 16 mitogenomes newly sequenced and annotated. We compared the linear arrangements of genes along mitochondria across species. Then we conducted 13 analyses each under a different combination of character coding, partitioning scheme and heterotachy models, and assessed their performance in phylogenetic inference. Several hypothetical tree topologies were also tested. Mitogenomic structure comparison revealed that most species share the same gene order of putative ancestral pancrustacean pattern, while seven species from Onychiuridae, Poduridae and Symphypleona bear different levels of gene rearrangements, indicating phylogenetic signals. Tomoceroidea was robustly recovered for the first time in the presence of all its families and subfamilies. Monophyly of Onychiuroidea was supported using unpartitioned models alleviating LBA. Paronellidae was revealed polyphyletic with two subfamilies inserted independently into Entomobryidae. Although Entomobryomorpha has not been well supported, more than half of the analyses obtained convincing topologies by placing Tomoceroidea within or near remaining Entomobryomorpha. The relationship between elongate-shaped and spherical-shaped collembolans still remained ambiguous, but Neelipleona tend to occupy the basal position in most trees. This study showed that mitochondrial genomes could provide important information for reconstructing the relationships among Collembola when suitable analytical approaches are implemented. Of all the data refining and model selecting schemes used in this study, the combination of nucleotide sequences, partitioning model and exclusion of third codon positions performed better in generating more reliable tree topology and higher node supports than others.

## Introduction

Collembola (springtails) are numerically dominant microarthropods in most terrestrial environments, with more than 9000 species reported in the world [1]. They play roles in detrital food webs, and are important to soil ecosystems for modulating litter decomposition processes and forming soil microstructure [2–3]. However, regarding the basal phylogeny of Collembola, it has so far not been resolved. Generally, the basal classification of Collembola has two periods: (1) firstly two groups Arthropleona and Symphypleona *sensu lato* were recognised based on body shape and segmentation, the former contained species with generally elongated body shape and clearly divided body segments, while the later contained species with spherical body shape and fused segments [4–6]; (2) later the two-group system was replaced by a four-group system, in which the Arthropleona was divided into Poduromorpha and Entomobryomorpha mainly based on the status of prothoracic tergum, and Symphypleona *s*. *l*. was divided into Symphypleona *sensu stricto* and Neelipleona for the morphological differences in body and leg segments [1, 7–11]. To date, the second system has been widely accepted, but was seldom completely recovered by modern phylogenetic analyses, while the first system could also be completely or partially recovered in a few phylogenetic reconstructions.

The phylogenetic relationships within Collembola have been investigated by using either morphological characters or molecular markers in previous studies. D’Haese [9] performed a phylogenetic study based on D1 and D2 regions of *28S* rDNA for 55 Collembola species. In his study, Entomobryomorpha and Symphypleona *s*. *s*., as well as some families (e.g. Entomobryidae, Hypogastruridae, Neanuridae), were not monophyletic. In the following year, the same author conducted a morphological phylogenetic reconstruction based on 131 characters from 67 taxa [12]. In this study monophyly of four orders (Entomobryomorpha, Neelipleona, Poduromorpha, and Sympypleona) and most families were supported, except for Onychiuridae and Hypogastruridae. Later, several molecular studies based on partial or complete *18S* and *28S* rDNA also indicated the relationships within Collembola [13–17]. These studies often yielded similar results, but support for some taxa could change significantly depending on taxa sampled. Luan et al. [13] indicated monophyly of Arthropleona, Entomobryomorpha and Poduromorpha, and polyphyly of Symphypleona *s*. *s*. and Hypogastruridae. Similar results were repeated by Gao et al. [14], except that the later study supported monophyly of Symphypleona *s*. *s*. von Reumont et al. [16] supported the monophyly of Entomobryomorpha, Poduromorpha and Symphypleona *s*. *s*., but rejected Arthropleona. Both Gao et al. [14] and von Reumont et al. [16] did not support Symphypleona *s*. *l*., with Neelipleona separated from Symphypleona *s*. *s*. and occupying the basal branch of Collembola. However, the three aforementioned studies were focused on higher phylogeny of either basal Hexapoda [13–14] or whole Arthropoda [16], thus only limited collembolan species, including 10 in Luan et al. [13], 7 in Gao et al. [14] and 14 in von Reumond et al. [16]. In contrast, Xiong et al. [15] aimed to resolve phylogenetic relationships within Collembola and sampled 30 species belonging to 29 genera and 14 families. This study supported the monophyly of Poduromorpha, Symphypleona *s*. *s*. and Symphypleona *s*. *l*., while Entomobryomorpha was not supported, with Tomoceroidea unexpectedly closer to Poduromorpha. With a further enriched sampling of 54 species, Yu et al. [17] emphasized the monophyly of Tomoceridae, separated Neelipleona from Symphypleona *s*. *s*., and doubted again the monophyly of Tomoceroidea and its relationship with other Entomobryomorpha. However, considering morphological evidence and limitation of taxon sampling in molecular study, the authors suggested keeping the current taxonomic system. With different gene markers (*16S* rDNA, *COX1* and D1‒D2 regions of *28S* rDNA) and taxon sampling focused on Neelipleona and Symphypleona, Schneider et al. [18] and Schneider and D’Haese [19] revealed a topology different from most other studies, showing that Neelipleona was sister to Arthropleona, and Symphypleona was basal to them, while Entomobryidae and Isotomidae were both polyphyletic. Zhang et al. [20–21] reconstructed the phylogeny of Entomobryoidea with *COX1*, *16S*, *18S* and *28S*, and found that the family Paronellidae was split into several independent groups included in Entomobryidae. A review of these previous studies shows that different gene markers, taxon samplings and analytical methods can lead to distinct tree topologies reflecting relationships among main groups of Collembola. Overall, the phylogenetic relationships within Collembola are still unresolved to a large extent, with the main controversial points as following: (1) monophylies of some families and superfamilies, e.g. Hypogastruridae, Paronellidae, Onychiuroidea, Tomoceroidea; (2) relationship between Tomoceroidea and other groups; (3) relationship between elongate-shaped and spherical-shaped groups.

The insect mitochondrial genome is usually a compact circular molecule typically 15–18 kb in size. It contains 37 genes: 13 protein-coding genes (PCGs) encoding subunits from four of the five mitochondrial electron-transport chain complexes, and two ribosomal RNA (rRNA) genes and 22 transfer RNA (tRNA) genes involved in the translation of the PCGs [22]. Mitogenomes have become popular multi-loci molecular markers for phylogenetic studies as having advantages of maternal hereditary transmission and accelerated nucleotide substitution rates compared with nuclear markers [22–25], and have recently received extensive use because improved sequencing techniques have made it more efficiency and less costly to obtain the complete sequences [26]. Further, the variation in mitogenomes may be related to the adaption of environmental conditions [27–29]. However, the power of mitogenomes in phylogenetic reconstructions is still controversial. On one hand, genomic data can be organized into different datasets, particularly PCGs can be coded in the forms of nucleotide sequences (all codon positions or first two positions only), codons or amino acids, while the performances of different character coding are not unified [30–31]. On the other hand, heterotachous evolutionary processes of mitogenome sequences are known to mislead phylogenetic inference, and various site-heterogeneous models, such as CAT (classifies sites into categories, [32]) and GHOST (general heterogeneous evolution on a single topology, [33]), have been explored to improve the reliability of results. Moreover, partitioning of alignments is also a common approach to incorporating the heterogeneity, however, its positive effects on tree topology, branch-lengths and bootstrap supports are still debated [34]. As a result, various character coding, partitioning schemes and models have been extensively applied to reconstruct the mitochondrial phylogenetic trees in many insect groups [22], e.g. Hemiptera [35], Heteroptera [36], Psocodea [37], Hymenoptera [38–39], Coleoptera [40], and Lepidoptera [41]. Mitogenomic analyses have also been implemented to solve some phylogenetic and taxonomic problems of Collembola [42–43]. A recent study has reported so far most comprehensive phylogenetic analysis of Collembola based on mitogenomes, with 11 families sampled [44]. In the context of their taxon sampling, they revealed the structural diversity of collembolan mitogenomes, recovered the monophyly of all four orders as well as six families, and contributed to the time frame of collembolan evolution. However, the effectiveness of various alternative analytical approaches has not been sufficiently evaluated, and the position of several significant and most problematic taxa, e.g. Tomoceridae, Oncopoduridae and Paronellidae, remains pending further assessment.

To further understand the phylogeny of Collembola, we have newly sequenced and annotated the mitogenomes of 16 collembolan species. Firstly, we identified and compared the alternative gene arrangements observed along the mtDNA of different taxa. Then, with the enlarged dataset, we performed a range of analyses based on different character coding, partitioning and site-heterogeneous models to address the phylogenetic problems within Collembola mentioned above. Finally, we compared different analytical approaches and assessed the effectiveness of mitogenomic analyses for reconstructing the phylogeny of Collembola.

## Materials and methods

### Taxon sampling

A total of 31 species representing main groups of all four orders of Collembola were selected for the phylogenetic reconstructions (Table 1 and S1 Table). Five families and nine subfamilies were newly introduced to mitophylogenomic analyses in this study, including elongate-shaped Paronellidae (Salininae and Paronellinae), Oncopoduridae, Tomoceridae (Lepidophorellinae and Tomocerinae), Pachyotominae, Heteromurinae and Entomobryinae, and spherical-shaped Katiannidae, Sminthurididae, Sphyrothecinae and Ptenothricinae. Complete and partial mitogenomes of 16 species were originally sequenced in this study, while data of the other 15 Collembola and three outgroups (two Diplura and one Microcoryphia species) were retrieved from the NCBI database. For newly sequenced species, specimens were collected with aspirators or Berlese funnels and preserved in 99% ethanol before morphological examination and DNA extraction. All specimens were morphologically identified to species level before DNA extraction. No permits were required for our collection.

**Table 1:** Information of collembolan species used in this study.

GenBank nos.	Order	Family	Subfamily	Species	PCGs	Genes	Size (bp)
MK014212	Entomobryomorpha	Entomobryidae	Entomobryinae	*Sinella curviseta* Brook, 1882	13	37	14,840
MK431895	Entomobryomorpha	Entomobryidae	Heteromurinae	*Dicranocentrus wangi* Ma & Chen, 2007	13	37	14,883
MK431900	Entomobryomorpha	Entomobryidae	Lepidocyrtinae	*Lepidocyrtus fimetarius* Gisin, 1964	13	37	14,698
KT985987	Entomobryomorpha	Entomobryidae	Orchesellinae	*Orchesella cincta* Linnæus, 1758	13	37	15,728
EU016195	Entomobryomorpha	Entomobryidae	Orchesellinae	*Orchesella villosa* von Linné, 1767	13	37	14,924
MK431896	Entomobryomorpha	Paronellidae	Paronellinae	*Cyphoderus albinus* Nicolet, 1842	13	37	14,836
MK409685	Entomobryomorpha	Paronellidae	Salininae	*Salina celebensis* (Schäffer, 1898)	13	37	14,788
NC_010533	Entomobryomorpha	Isotomidae	Anurophorinae	*Cryptopygus antarcticus* Willem, 1901	13	37	15,297
KX863671	Entomobryomorpha	Isotomidae	Anurophorinae	*Cryptopygus terranovus* (Wise, 1967)	13	37	15,352
KU198392	Entomobryomorpha	Isotomidae	Anurophorinae	*Folsomia candida* Willem, 1902	13	37	15,147
NC_024155	Entomobryomorpha	Isotomidae	Isotominae	*Folsomotoma octooculata* (Willem, 1901)	13	37	15,338
MK423967	Entomobryomorpha	Isotomidae	Pachyotominae	*Paranurophorus simplex* Denis, 1929	11	28	9,518
MK431894	Entomobryomorpha	Oncopoduridae		*Oncopodura yosiiana* Szeptycki, 1977	13	37	14,808
MK431898	Entomobryomorpha	Tomoceridae	Lepidophorellinae	*Novacerus tasmanicus* (Womersley, 1937)	13	36	15,518
MK423966	Entomobryomorpha	Tomoceridae	Tomocerinae	*Tomocerus qinae* Yu, Yao & Hu, 2016	13	37	15,045
MK409686	Poduromorpha	Hypogastruridae		*Ceratophysella communis* (Folsom, 1898)	13	37	15,331
AY191995	Poduromorpha	Hypogastruridae		*Gomphiocephalus hodgsoni* Carpenter, 1908	13	37	15,075
EU084034	Poduromorpha	Neanuridae	Neanurinae	*Bilobella aurantiaca* (Caroli, 1912)	13	37	16,312
EU124719	Poduromorpha	Neanuridae	Frieseinae	*Friesea grisea* (Schäffer, 1891)	13	37	15,442
NC_006074	Poduromorpha	Onychiuridae	Onychiurinae	*Thalassaphorura orientalis* Stach, 1964	13	34	12,984
MK423968	Poduromorpha	Onychiuridae	Onychiurinae	*Thalassaphorura encarpata* (Denis, 1931)	13	37	15,213
NC_002735	Poduromorpha	Onychiuridae	Tetrodontophorinae	*Tetrodontophora bielanensis* (Waga, 1842)	13	37	15,455
MK431897	Poduromorpha	Tullbergiidae	Mesaphorurinae	*Mesaphorura yosii* (Rusek, 1967)	13	37	14,833
NC_006075	Poduromorpha	Poduridae		*Podura aquatica* Linnæus, 1758	13	34	13,809
MK431893	Neelipleona	Neelidae		*Neelides* sp.	13	34	13,858
MK423965	Symphypleona	Dicyrtomidae	Ptenothricinae	*Ptenothrix huangshanensis* Chen & Christiansen, 1996	13	37	15,152
MK423969	Symphypleona	Katiannidae		*Sminthurinus signatus* (Krausbauer, 1898)	7	20	5,459
MK423964	Symphypleona	Sminthurididae		*Sminthurides bifidus* Mills, 1934	13	35	14,161
KY618680	Symphypleona	Bourletiellidae		*Bourletiella arvalis* (Fitch, 1862)	13	37	14,794
NC_010536	Symphypleona	Sminthuridae	Sminthurinae	*Sminthurus viridis* (Linnæus, 1758)	13	37	14,817
MK431899	Symphypleona	Sminthuridae	Sphyrothecinae	*Lipothrix lubbocki* (Tullberg, 1872)	13	37	15,141

### DNA extraction, amplification and sequencing

DNA was extracted using a QIAamp DNA Micro Kit (QIAGEN GmbH, Shanghai, China). Extractions were performed non-destructively for further morphological examination and identification of the specimens. DNA concentration was measured by Qubit 3.0 using Q33230 Qubit™ 1X dsDNA HS Assay Kit. Mitogenome amplification for less than 50 ng DNA was performed using REPLI-g Single Cell Kit. Each library was sequenced with an insert size of 350 bp on HiSeq X Ten platform (Tianjin Novogene Bioinformatics Technology Co., Ltd, China) generating 150 bp paired-end reads.

The *COX1* was used as a seed sequence for our mitochondrial assembly. Primers for *COX1* were LCO1490/HCO2198 commonly used for Metazoa [45]. Amplification volume and procedure followed Zhang et al. [46]. All PCR products were checked on a 1% agarose gel. Successful products were purified and sequenced in both directions by Tsingke (Beijing, China) on ABI 3730XL DNA Analyser (Applied Biosystems). Sequences were assembled in Sequencher 4.5 (Gene Codes Corporation, Ann Arbor, USA), blasted in GenBank and checked for possible errors, then were preliminarily aligned using MEGA 7.0 [47]. Alignments were checked and corrected manually, with a final 658 bps alignment.

### Mitogenome assembly and annotation

All mitogenomes were assembled with NOVOPlasty v2.7.0 [48] using *COX1* sequence as the initial seed. Mitochondrial gene annotations were performed using MITOS web server [49] and tRNAs gene limits were rechecked with tRNAscan-SE [50]. The mtDNA sequences were deposited in GenBank (Table 1 and S1 Table).

### Phylogenetic analysis

Alignments of *16S* rRNA (*rrnL*), *12S* rRNA (*rrnS*) and amino-acid sequences of each PCG were conducted by MAFFT v.7.394 [51] with an accurate option L-INS-I, and then an automated alignment trimming (-automated1) was performed by trimAL v.1.4 [52] for removing gap-only and ambiguous-only positions. After that, we generated codon-based nucleotide sequence alignments of 13 PCGs by trimAL v.1.4 with the option -backtrans based on trimmed amino-acid sequences and unaligned nucleotide sequences of each gene. The final concatenated supermatrices were performed by FASconCAT-G v1.04 [53] as: (A1) nucleotide sequences of 13 PCGs (13fna), (A2) nucleotide sequences of 13 PCGs and two rRNA genes (15fna), (A3) amino acid sequences of 13 PCGs (13faa).

For PCGs in the supermatrices A1 and A2, we analyzed nucleotide sequences by using either all codon positions or excluding third codon positions (site 1+2) to evaluate the effect of saturation of the third codon positions of nucleotide substitutions. All partition and substitution models on supermatrices A1, A2 and A3 were selected using ModelFinder [54]. In addition, we also inferred trees from supermatrix A1 under the codon model (CODON5). Considering the heterogeneous evolution, GHOST model were applied in all supermatrices with nucleotide- (GHOST_GTR), codon- (GHOST_GY), and amino acid- (GHOST_LG and GHOST_mtART) models, respectively. Finally, we constructed trees from supermatrix A3 under posterior mean site frequency (PMSF) model [55], a variant of PhyloBayes’ CAT model, with 20 amino-acid profile categories under both LG and mtART exchange rate matrices (option: -m LG+C20+F+G and -m mtART+C20+F+G). Therefore, in total 13 analyses were performed as follows: (1) 13 fna_GHOST_GTR; (2) 13 fna_CODON5_GHOST_GY; (3) 13fna_13partition; (4) 13fna_13partition_CODON5; (5) 13fna_13partition_site1+2; (6) 15fna_ GHOST_GTR; (7) 15fna_15partition; (8) 15fna_13PCGs (site1+2)_2rrn (2partition); (9) 13faa_GHOST_LG; (10) 13faa_GHOST_mtART; (11) 13faa_PMSF_LG; (12) 13faa_PMSF_mtART; (13) 13faa_13partition. The maximum-likelihood (ML) trees were inferred and ultrafast bootstrap [56] with 1,000 replicates were performed in IQ-TREE v1.6.3 [57]. Nodes with a bootstrap value of minimum 95 were considered well-supported in the analyses.

### Tree topology comparison

Tree topology comparison on constraining monophyly was performed by using the RELL approximation method [58]. Seven hypotheses were proposed based on current ambiguities of collembolan phylogeny: (A) best tree without any constrains; (B) Tomoceridae + Oncopoduridae; (C) Neelipleona + Symphypleona *s*. *s*.; (D) Tomoceroidea + (Entomobryoidea + Isotomidae); (E) Tomoceroidea + Poduromorpha; (F) Tullbergiidae + Onychiuridae; (G) Symphypleona *s*. *s*. + Entomobryomorpha. Approximately unbiased (AU) test [59], bootstrap proportion (BP, [58]), expected likelihood weight (ELW, [60]), Kishino-Hasegawa (KH) test [61], Shimodaira-Hasegawa (SH) test [62], weighted KH (WKH) and weighted SH (WSH) tests were performed in IQ-TREE v.1.6.3 with the options: -au, -zb and -zw. The number of RELL replicates was specified to 10,000.

## Results

### Characteristics of collembolan mitochondrial genomes

**Fig 1:**
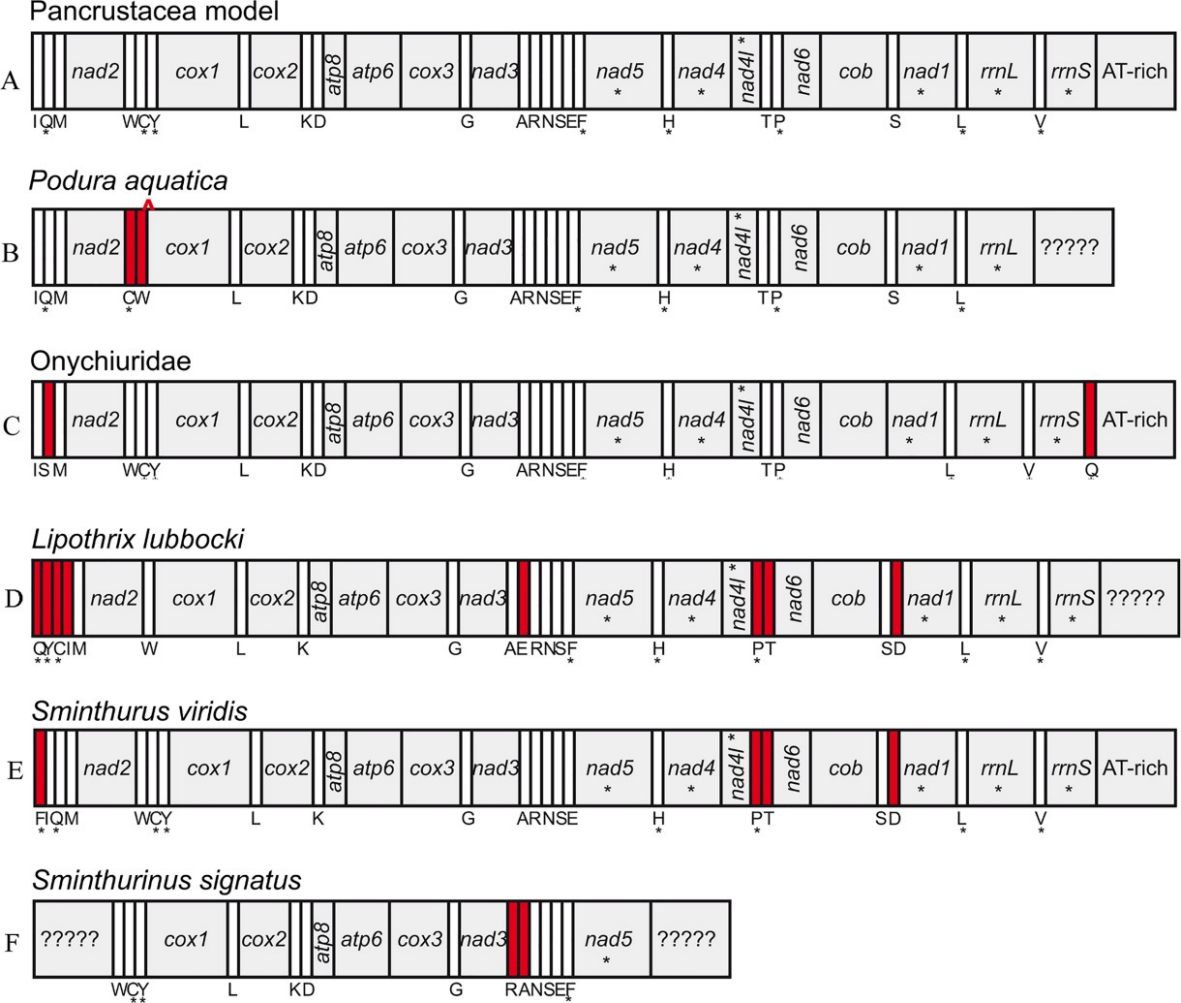
Comparison of linear arrangements of genes on mitochondrial genomes of collembolan species in this study. A. Pancrustacea (putative ancestral) model in most species. B. *Podura aquatica*. C. Onychiuridae. D. *Lipothrix lubbocki*. E. *Sminthurus viridis*. F. *Sminthurinus signatus*. The transcriptional direction of mitochondrial genes is from left to right, the asterisk (*) below the genes indicates the opposite direction, the question marks indicates the unknown genes, the rearrangements are marked in red, the inverted triangle indicates the deletion of gene.

Complete and partial mitogenomes newly sequenced range from 5,459 bp to 16,312 bp in size (Table 1). The mitogenomes of 24 species possessed the putative ancestral Pancrustacea gene order, while gene rearrangements were observed in seven species with deletion, inversion or translocation of tRNA genes (Fig 1, S2 Table). *Podura aquatica* Linnæus, 1758 (Poduridae) has the deletion of *trnF* between *trnC* and *cox1*, and the inversion between *trnC and trnW*. Three species of Onychiuridae have translocation of *trnS (uga)* and *trnQ*. Three species of Symphypleona have different gene rearrangements. *Lipothrix lubbocki* (Tullberg, 1872) (Sminthuridae: Sphyrothecinae) have the highest level of tRNA rearrangements, including inversions between *trnQ* and *trnI*, between *trnP* and *trnT*, and between *trnC* and *trnY*, and translocations of *trnC*, *trnY*, *trnD*, and *trnE*. *Sminthurus viridis* (Linnæus, 1758) (Sminthuridae: Sminthurinae) have the inversion between *trnP* and *trnT*, and the translocation of *trnD* and *trnF*. *Sminthurinus signatus* (Krausbauer, 1898) (Katiannidae) have the inversion between *trnR* and *trnA*.

### Phylogenetic inference

**Fig 2:**
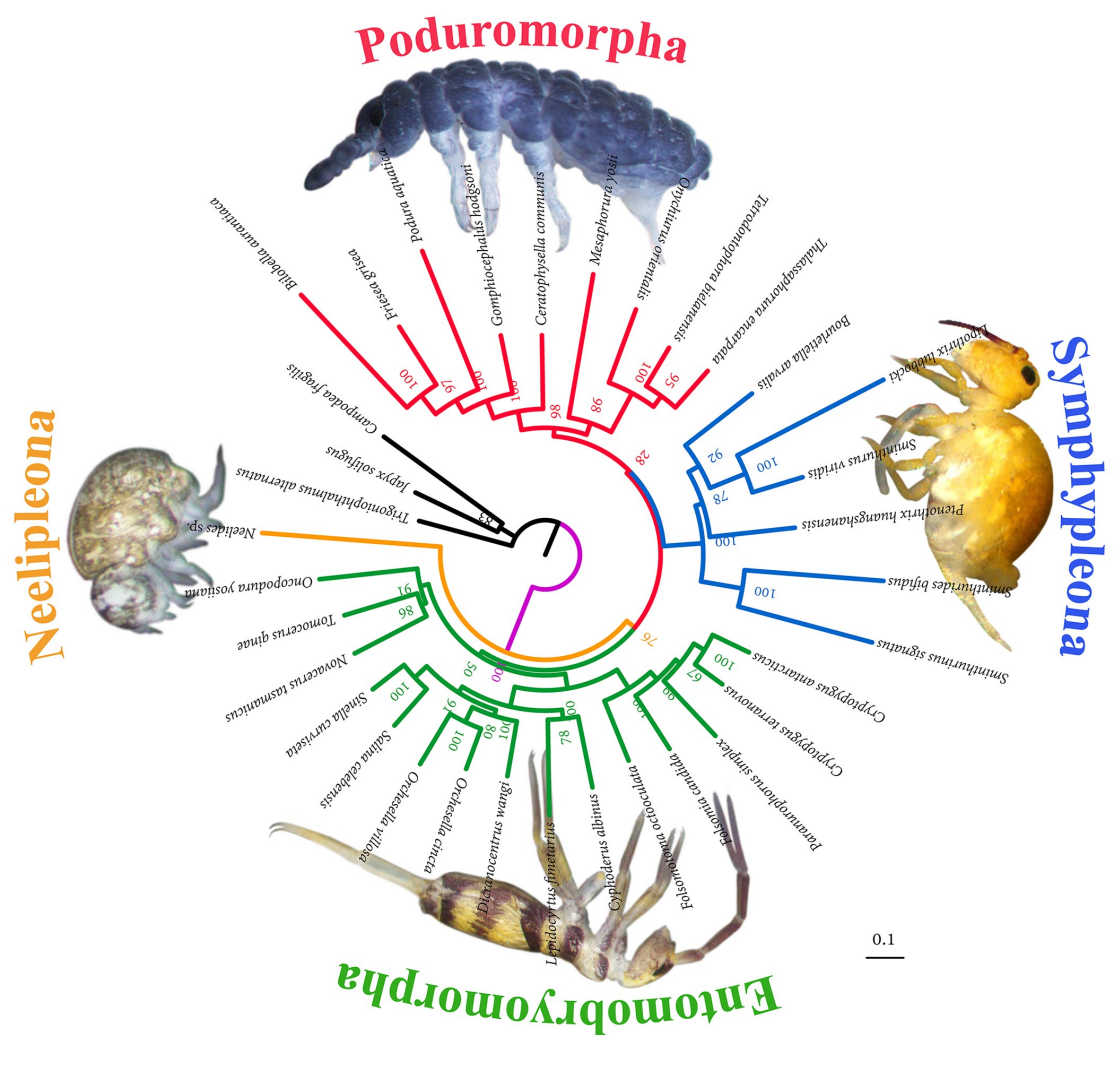
Maximum likelihood phylogenetic tree inferred from partitioned nucleotide sequences of 15 genes, with third codon position excluded (15fna_13PCGs (site1+2)_2rrn (2partition)). Bootstrap support values are shown in the nodes.

Among all 13 analyses using different character coding, partitioning scheme and heterotachy models, the combination of 15fna_13PCGs (site1+2)_2rrn (2partition), using nucleotide sequences of 15 genes, partitioning model and exclusion of third codon positions, performed better in generating more plausible tree topology and higher node supports, (Fig 2), which is generally in congruence with external sources of evidence such as morphology and/or other molecular studies (see Discussion Section for details). In this best-resolved tree, the monophyly of most well-defined families and subfamilies is recovered with high support values (support>80), with the exception of Hypogastruridae, Paronellidae and Entomobryidae. Within Hypogastruridae, *Gomphiocephalus hodgsoni* is sister to the clade of Neanuridae + Poduridae, and *Ceratophysella communis* is further sister to them. Two species of Paroneliidae are included in Entomobryidae. Moreover, all three orders represented by more than one species are recovered, despite that Entomobryomorpha does not receive high support (support = 50). Thus this result is generally satisfactory because it conforms well with currently accepted classification system of Collembola based on morphological examination (see Discussion Section for details). Nevertheless, considering that the controversial phylogenetic relationship within Collembola has not been settled to a large extent, the incongruence between analyses may provide alternative valuable hypotheses which are to be tested by further studies, therefore, results of all analyses are thoroughly listed, compared and discussed below.

The phylogenetic trees calculated from all supermatrices (A1, A2 and A3) strongly supported the monophyly of Symphypleona *s*. *s*. Symphypleona *s*. *s*. clustered with Entomobryoidea + Isotomidae by analyses of 13fna_GHOST_GTR, 13fna_13partition, 15fna_GHOST_GTR and 15fna_15partition, however, with low to medium supports (33.1, 84, 34.5, 79, respectively). The basal position of Neelipleona was strongly supported in most analyses, except 13faa_GHOST_mtART and 13faa_PMSF_LG clustered Symphypleona *s*. *s*. and Neelipleona together (support = 73.9 and 76.1, respectively).

Poduromorpha was supported in most analyses, but Tullbergiidae was clustered with Neelipleona in 13fna_GHOST_GTR (support = 78.1), 13fna_CODON5_GHOST_GY (support = 69.5), 13faa_GHOST_LG (support = 68.3) and 13faa_PMSF_mtART (support = 95.2). In eight other analyses, Tullbergiidae was clustered with Onychiuridae, and six of them were well supported (support>80). The monophyly of Onychiuridae was well supported in all analyses, but the subfamily Onychiurinae is not monophyletic. Neanuridae was recovered in most analyses, with Poduridae as sister group, and basal to them were two species of Hypogastruridae usually forming a paraphyletic group.

The status of Entomobryomorpha was highly incongruent between analyses. Entomobryoidea and Isotomidae were both recovered and clustered together with high supports in all analyses (support>95). Tomoceroidea was highly supported in most analyses (support>90 in 10 analyses), and it was clustered with Entomobryoidea + Isotomidae, forming Entomobryomorpha in 13fna_CODON5_GHOST_GY (support = 49.8), 13fna_13partition_CODON5 (support = 30.9), 13fna_13partition_site1+2 (support = 51), 15fna_13PCGs(site1+2)_2rrn (2partition) (support = 50) and 13faa_13partition(support = 29), but with only low supports. In 13fna_GHOST_GTR and 15fna_GHOST_GTR, Tomoceroidea was basal to the branch containing Symphypleona + (Entomobryoidea + Isotomidae) (support = 72.9 and 41.2, respectively), while in the other six analyses it was clustered with Poduromorpha. Monophyly of Tomoceridae was recovered in eight analyses (with high support except in 13faa_GHOST_LG with support = 18.5), while 13fna_GHOST_GTR, 13fna_13partition, 15fna_GHOST_GTR and 15fna_15partition clustered Tomocerinae with Oncopoduridae (support = 31.8, 83, 27.6 and 86, respectively), and 13fna_CODON5_GHOST_GY clustered Lepidophorellinae with Oncopoduridae (support = 9.6). Paronellidae was polyphyletic in all analyses, with Salininae always clustered with Entomobryinae, and Paronellinae often clustered with Lepidocyrtinae.

**Fig 3:**
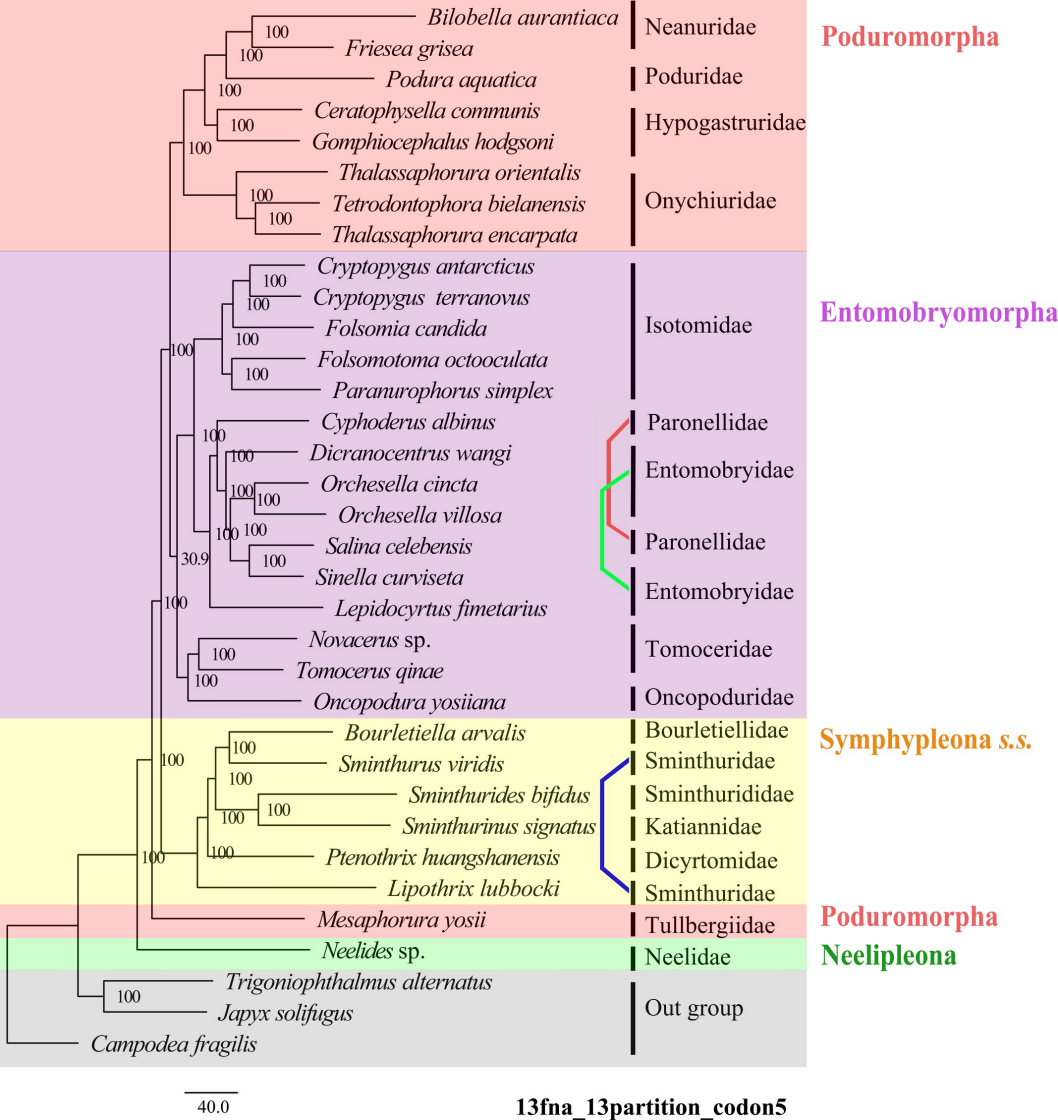
Maximum likelihood phylogenetic tree inferred from partitioned nucleotide sequences of 13 PCGs under codon 5 model (13fna_13parition_CODON5). Bootstrap support values are shown in the nodes. Each coloured line linked a same taxon in current taxonomic system but separated in different branches.

**Fig 4:**
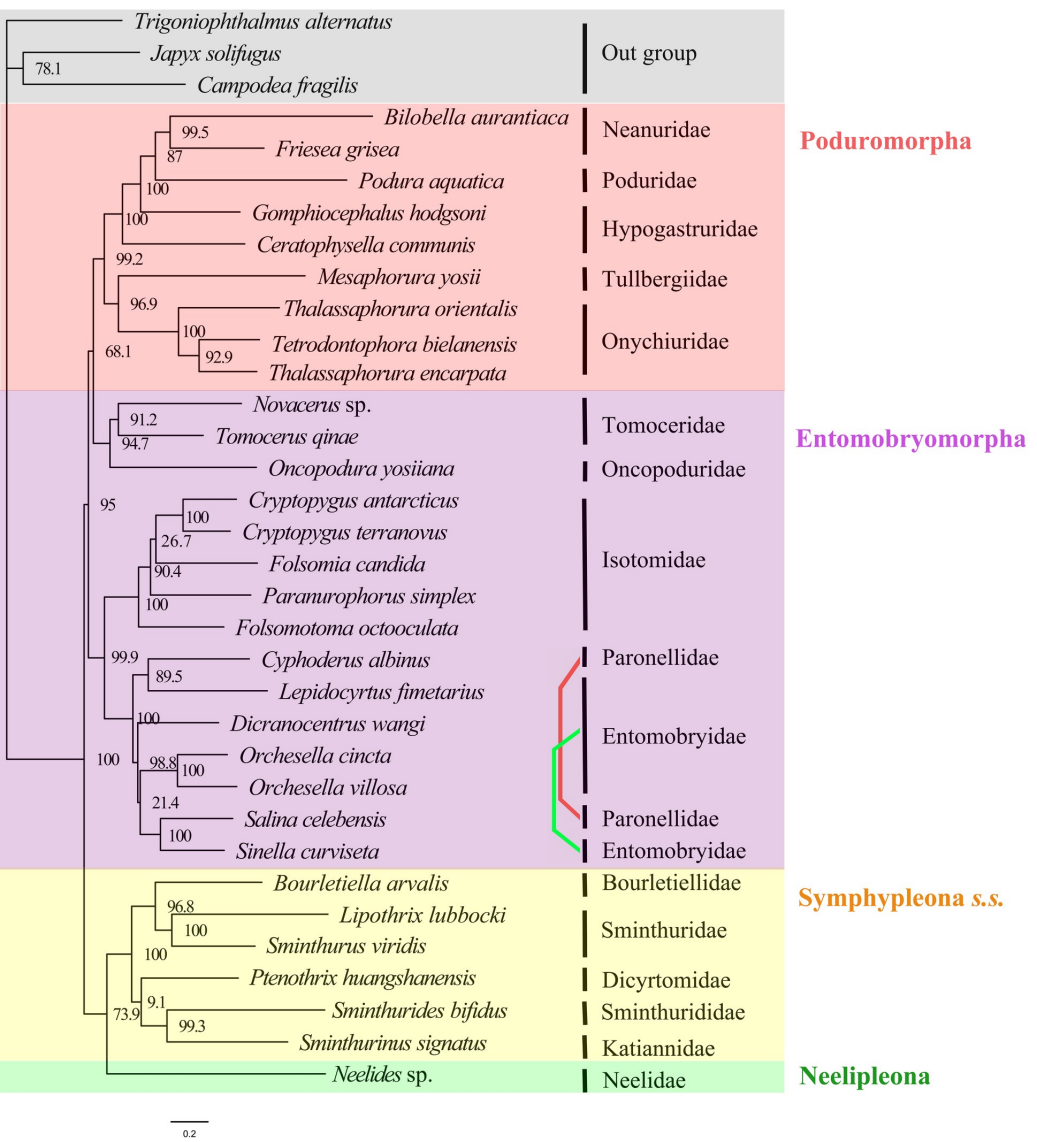
Maximum likelihood phylogenetic tree inferred from amino acid sequences of 13 PCGs under GHOST_mtART model (13faa_GHOST_mtART). Bootstrap support values are shown in the nodes. Each coloured line linked a same taxon in current taxonomic system separated in different branches.

Support values for major clades in all analyses were shown in Table 2. Results of two analyses were selected to represent main variations in tree topologies (Figs 3 and 4), while results of other analyses were deposited in S1 Fig.

**Table 2:** Support values for the major clades recovered by different datasets and analytical approaches. “N” means this was not supported.

	13fna					15fna			13faa				
	GHOST _GTR	CODON5_GHOST_GY	13partition	13partition_CODON5	13partition_site1+2	GHOST _GTR	15partition	13PCGs(site1+2)_2rrn (2partition)	GHOST_LG	GHOST _mtART	PMSF _LG	PMSF_mtART	13partition
Poduromorpha	N	N	83.0	N	95.0	99.9	95.0	98.0	N	99.2	98.9	N	51.0
Symphypleona	100.0	100.0	100.0	100.0	100.0	100.0	97.0	100.0	100.0	100.0	100.0	100.0	100.0
Entomobryomorpha	N	49.8	N	30.9	51.0	N	N	50.0	N	N	N	N	29.0
Symphypleona *s*. *l*.	N	N	N	N	N	N	N	N	N	73.9	76.1	N	N
Neelipleona at base	100.0	98.8	100.0	100.0	100.0	100.0	100.0	100.0	100.0	N	N	24.0	100.0
Tomoceroidea + Poduromorpha	N	N	72.0	N	N	N	68.0	N	21.5	68.1	91.1	78.4	N
Tullbergiidae + Onychiuridae	N	N	82.0	N	96.0	98.0	93.0	98.0	N	96.9	31.4	N	52.0
Entomobryoidea + Isotomidae	100.0	100.0	100.0	100.0	100.0	100.0	97.0	100.0	100.0	99.9	100.0	100.0	100.0
Symphypleona + (Entomobryoidea+Isotomidae)	33.1	N	84.0	N	N	34.5	79.0	N	N	N	N	N	N
Poduridae + Neanuridae	82.0	99.6	87.0	100.0	96.0	72.2	91.0	97.0	98.5	87.0	87.9	74.0	N
Tomoceridae	N	N	N	100.0	81.0	N	N	86.0	18.5	91.2	85.1	88.5	86.0
Hypogastruridae	N	N	N	100.0	N	N	N	N	N	N	N	N	N
Paronellidae	N	N	N	N	N	N	N	N	N	N	N	N	N
Sminthuridae	100.0	100.0	100.0	N	100.0	100.0	100.0	100.0	100.0	100.0	100.0	100.0	100.0
Onychiurinae	N	N	77.0	N	N	N	N	N	N	N	N	N	N

### Tree topology tests

Most hypotheses were not rejected by tree topology tests in most analyses (Table 3 and S3 Table). However, in 13fna_13partition_CODON5 most hypotheses were significantly rejected except for the best tree without any constrains. In addition, hypothesis (C) Neelipleona + Symphypleona *s*. *s*. was significantly rejected in the analyses of 15fna_GHOST_GTR and 15fna_15partition.

**Table 3:** Results of tree topology tests of the analyses 13fna_13parition_CODON5 and 13faa_GHOST_mtART. Seven hypothetical topologies were (A) best tree without any constrains; (B) Tomoceridae + Oncopoduridae; (C) Neelipleona + Symphypleona s. s.; (D) Tomoceroidea + (Entomobryoidea + Isotomidae); (E) Tomoceroidea + Poduromorpha; (F) Tullbergiidae + Onychiuridae; (G) Symphypleona s. s. + Entomobryomorpha.

Hypotheses	logL	deltaL	bp-RELL	p-KH	p-SH	p-WKH	p-WSH	c-ELW	p-AU
**13fna_13parition_CODON5**									
**A**	-226076,295	0,000	0.9684 +	0.9710 +	1.0000 +	0.9710 +	0.9997 +	0.9684 +	0.9818 +
**B**	-226439,654	363,359	0.0000 -	0.0000 -	0.0000 -	0.0000 -	0.0000 -	0.0000 -	0.0000 -
**C**	-226591,485	515,191	0.0000 -	0.0000 -	0.0000 -	0.0000 -	0.0000 -	0.0000 -	0.0000 -
**D**	-226159,904	83,610	0.0291 -	0.029 -	0.2168 +	0.0290 -	0.1332 +	0.0291 -	0.0295 -
**E**	-226325,936	249,641	0.0000 -	0.0000 -	0.0000 -	0.0000 -	0.0000 -	0.0000 -	0.0000 -
**F**	-226685,661	609,366	0.0000 -	0.0000 -	0.0000 -	0.0000 -	0.0000 -	0.0000 -	0.0000 -
**G**	-226236,015	159,721	0.0025 -	0.0035 -	0.0216 -	0.0035 -	0.0156 -	0.0025 -	0.0029 -
**13faa_GHOST_mtART**									
**A**	-116116,632	0,047	0.0000 -	0.4240 +	0.8651 +	0.4240 +	0.9231 +	0.0294 +	0.5346 +
**B**	-116116,586	0,001	0.0018 -	0.4468 +	0.9604 +	0.4468 +	0.9457 +	0.0307 +	0.7470 +
**C**	-116116,639	0,054	0.0093 -	0.4160 +	0.8695 +	0.3319 +	0.9636 +	0.0292 +	0.7458 +
**D**	-116116,585	0,000	0.0196 +	0.5532 +	1.0000 +	0.5772 +	0.9632 +	0.0307 +	0.7241 +
**E**	-116116,587	0,002	0.0374 +	0.3935 +	0.9447 +	0.3935 +	0.9060 +	0.0307 +	0.7467 +
**F**	-116116,632	0,047	0.0648 +	0.4228 +	0.8619 +	0.4228 +	0.9494 +	0.0294 +	0.6709 +
**G**	-116139,142	22,557	0.0144 +	0.1280 +	0.1313 +	0.1280 +	0.3050 +	0.0226 +	0.1397 +

deltaL: logL difference from the maximal logl in the set. bp-RELL: bootstrap proportion using RELL method [54]. p-KH: p-value of one sided Kishino-Hasegawa test [57]. p-SH: p-value of Shimodaira-Hasegawa test [58]. p-WKH: p-value of weighted KH test. p-WSH: p-value of weighted SH test. c-ELW: Expected Likelihood Weight [56]. p-AU: p-value of approximately unbiased (AU) test [55]. Plus signs denote the 95% confidence sets. Minus signs denote significant exclusion. All tests performed 10000 resamplings using the RELL method.

Results of the topology tests for two selected trees were shown in Table 3, while the others were deposited in S3 Table.

## Discussion

### Mitochondrial gene rearrangements

Similar to nucleotide and amino acid sequences, the arrangements of genes along mitochondrial chromosome may also reflect evolutionary history and phylogenetic relationships [63–64]. Among 31 species in our study, seven species of four families, including four species of Poduromorpha (three Onychiuridae and one Poduridae) and three species of Symphypleona (two Sminthuridae and one Katiannidae), have different mitochondrial gene arrangements. All these gene rearrangements occur in tRNA genes but none in PCGs, as also commonly found in insects [22]. Synapomorphic gene rearrangements were observed on the familial level, which is in line with previous studies in Collembola and many groups of insects [22, 44]. Three species of Onychiuridae have identical translocation of the same genes as a synapomorphy. Besides, the partial sequence of mitogenome of *Tullbergia mixta* Wahlgren, 1906 (Tullbergiidae: Tullbergiinae), also has the same translocation of *trnS (uga)* (GenBank accession number KF982833.1), however, no such rearrangement was found in another tullbergiid species, *Mesaphorura yosii* Rusek, 1967 from another subfamily (Mesaphorurinae). Similarly, two species from two subfamilies of Sminthuridae share the translocation of *trnD* and inversion between *trnP* and *trnT* as synapomorphies, but have different rearrangements for other genes. *Podura aquatica* lives almost exclusively on the surface of freshwater bodies, thus is ecologically remote from closely related neanurids and hypogastrurids which are mostly soil dwellers. Our results suggest the rearrangement of mitochondrial genes is taxon-specific and may reflect certain evolutionary events in Collembola. However, most families are represented by only a few species in available mitogenome databases, thus a thorough comparative study is not feasible until more species have been sequenced.

### Basal phylogeny of Collembola

Of the four orders of Collembola, one, the Symphypleona *s*. *s*., was strongly supported by our results, which is in line with most previous studies based on morphological and multi-locus molecular analyses [12, 15, 17]. This order is well characterized by the spherical body shape and can be distinguished from another spherical-shaped order Neelipleona by numerous characters such as the ratio between thoracic and abdominal segments, status of sensory organs on body and length of antennae [65]. Only two analyses clustered Symphypleona *s*. *s*. and Neelipleona together, while other analyses all indicated Symphypleona *s*. *s*. is more related to non-spherical groups (Arthropleona), and Neelipleona is basal to them. Additionally, the topology test also rejected Symphypleona *s*. *l*. hypothesis for the trees based on 15 nucleotide sequences using either partitioning or GHOST model. Four analyses have retrieved the topology of (Entomobryoidea + Isotomidae) + Symphypleona *s*. *s*., which is in line with the result of Leo et al. [44], and is supported by their morphological similarities such as reduction of prothoracic tergum, presence of abdominal bothriotricha and chaetotaxy on legs. But this topology was not strongly supported in our analyses. The ambiguity may be caused by the lack of intermediate forms between spherical and elongated groups in our dataset. Compared to the five families of Symphypleona *s*. *s*. included in this study, a rarely discovered but widespread family Mackenziellidae, has relatively elongated body shape and less fused body segments, which is considered probably more primitive in this order [66] (although also speculated as secondarily derived from globular ancestor by Fjellberg [67]), and is expected to be able to enhance the link between Symphypleona *s*. *s*. and other Collembola in further studies.

Poduromorpha was well supported by our analyses, except the unexpected position of Tullbergiidae in a few analyses. The unusual grouping of Tullbergiidae with Neelipleona was most probably caused by long branch attraction (LBA) [68–69]. In morphology, the Tullbergiidae is far remote from Neelipleona but closest to Onychiuridae, which was supported by six of our analyses with high support value (Table 2). However, the sister relationship between Onychiuridae and Tullbergiidae is still controversial. Similarly, Leo et al. also recovered different topology for the position of Tullbergiidae by using datasets either including or excluding the third codon positions [44]. Resolving this problem may require further assessment involving other assumed members of the superfamily Onychiuroidea, such as Odontellidae Massoud, 1967 and Pachytullbergiidae Stach, 1954. Moreover, the deep genetic divergence indicated by relatively long branch lengths supported the separation between Tullbergiidae and Onychiuridae, and indicated these two apparently similar groups may have undergone different selective pressures affecting the mitochondrial genomes [43]. From an eco-morphological aspect, although both groups are usually considered as euedaphic lifeform [3, 70], most tullbergiids have a smaller body size and more slender body shape than onychiurids. This indicates they are more adapted to finer pores in deep soil, while in epedaphic habitats (litters, mosses), onychiurids are more frequent than tullbergiids. Additional comparative studies are required to address how habitat preference of Collembola could have influenced their mitogenomes.

The Entomobryomorpha was recognised by only five analyses with low to medium support. Among this order, Isotomidae and Entomobryoidea were clustered together with high support in all analyses, which is in line with most previous studies [14–17, 44]. The problem in the position of the Tomoceroidea still exists, however, compared to other molecular studies focusing on the phylogeny of Collembola or Tomoceroidea [9, 15, 17], the present study for the first time clustered Tomoceroidea with other Entomobryomorpha. Similar results were reported by more comprehensive phylogeny of Arthropoda [16] and Insecta [71]. Among the analyses which did not retrieve Entomobryomorpha, two of them using nucleotide datasets with heterotachy models still indicated the basal position of Tomoceroidea within the branch containing Symphypleona *s*. *s*. and Entomobryomorpha. On morphological grounds, the Tomoceroidea is more similar to Entomobryidae and Isotomidae than any other groups [17], especially in the form of the prothoracic tergum and appendages. From an ecological aspect, life form and trophic niche also indicate Tomoceroidea closer to other Entomobryomorpha [3, 72]. Therefore, the previous hypothesis should be kept that the Tomoceroidea is a branch of Entomobryomorpha splitting early from the main trunk [17], resulting in considerable genetic divergences.

### Infra-ordinal phylogeny

Since more species and groups have been added, our study also provided information in infra-ordinal level phylogeny of Collembola. Several current familial and superfamilial groupings were strongly supported in most analyses, including Onychiuridae, Neanuridae, Isotomidae, Entomobryoidea and Sminthuridae, which have also been well defined by morphological characters, such as status of prothorax, pseudocelli, antennal sensory organs, body segment ratios, mouthparts and chaetotaxy [73–74]. Paronellidae was revealed to be polyphyletic, with two subfamilies, Salininae and Paronellinae, related to two other subfamilies Lepidocyrtinae and Entomobryinae of Entomobryidae, respectively. This result coincided with the findings of Zhang et al. [21–22] based on both molecular and morphological evidence, and supported the division between Salininae and Paronellinae [73]. We found the Hypogastruridae was paraphyletic and Tetrodontophorinae was within Onychiurinae. These findings were also in conflict with traditional morphological classification, but similar to the results of previous molecular studies [9, 15, 17, 44, 75]. These problematic families should receive thorough revisions in future studies.

The present study, for the first time, strongly supports the monophyly of Tomoceroidea when all three familial/subfamilial taxa were present. However, the relationships between the three groups was not determined, as seven analyses supported the monophyly of Tomoceridae, four analyses supported Tomocerinae + Oncopoduridae, and two analyses supported Oncopoduridae + Lepidophorellinae. Tomoceridae was recovered in another study using 18S and 28S genes [17], but the species of Lepidophorellinae included in two studies belong to two morphologically distinct genera and tribes. *Lepidophorella* (tribe Lepidophorellini) used in the previous study has a curved dens and short falcate mucro, while *Novacerus* (tribe Novacerini) used in the present study has a straight dens and elongated multi-dentate mucro. Relationship between the two tribes is so far unclear. Number of eyes and ratio of the length of antennal segments indicate Novacerini is more similar to Lepidophorellini, but the morphology of the jumping organ may group Novacerini with Tomocerinae. However, despite the significant difference in body size, Novacerini and Oncopoduridae are also similar in antennal ratio, presence of postantennal organs (unpublished data) and morphology of mucro. For example, the structure of mucro in *Novacerus* is more similar to that of Oncopoduridae (in particular *Harlomillsia*) than to that of Tomocerinae in arrangement and relative size of dorsal teeth. Therefore, on the basis of current progresses in morphological data and molecular phylogeny, an expected relationship within Tomoceroidea is probably (((Lepidophorellini + Tomocerinae) + Novacerini) + Oncopoduridae), which hypothesis requires further survey.

### Performance of different analytical methods

Both morphological evidence and previous molecular studies have strongly supported Tullbergiidae within Poduromorpha and probably the sister group of Onychiuridae [9, 12, 15, 17]. Therefore, although some analyses showed strong support for Tullbergiidae + Neelipleona, these results can be attributed to long-branch effects caused by model mis-specification [55]. Accordingly, implausible results were yielded by three analyses using PCG sequences with codon and GHOST models and two analyses using amino acids with GHOST_LG and PMSF_mtART models. In comparison, all three analyses using both protein-coding and rRNA genes (15fna) supported Tullbergiidae + Onychiuridae (Onychiuroidea) with high support values (support≥95). This is in line with other mitophylogenomic studies of insects showing that the inclusion of rRNA could improve resolution and nodal support [76–77], although rRNA may have higher levels of homoplasy than PCGs [76]. Two analyses using partitioned PCG sequences without codon model and one analysis using partitioned amino acid sequences also clustered Tullbergiidae with Onychiuridae, showing an advantage of partitioning method. This confirmed that algorithmically optimized partitioning schemes outperform unpartitioned analyses in phylogenetic inference due to better accommodation to the variation in substitution patterns among sites [34]. The other two analyses recovering Tullbergiidae + Onychiuridae were based on amino acids with GHOST_mtART and PMSF_LG models, suggesting the importance of selecting suitable exchange rate matrices under different heterotachy models.

Although Tomoceroidea has been previously clustered with Poduromorpha in some phylogenetic analyses based on a few rDNA markers [9, 15, 17], strong evidences from morphology [17, 78], ecology [3, 72], transcriptomes [71] and complete genomes [79] all support its closer relationship with other Entomobryomorpha than with Poduromorpha. Therefore, the topology of Tomoceroidea + Poduromorpha even with apparently robust supports might be considered incorrect. Accordingly, two analyses using partitioned nucleotide sequences excluding third codon positions and one analysis using partitioned amino acid sequences outperformed others among those also successfully recovering Onychiuroidea. Under lower selective pressures, the third codon positions are strongly affected by base compositional bias [22], thus are probably important source of homoplasy [76], which may result in artificial phylogenetic affinities. However, the inclusion of third codon positions do not consistently affect the topology and robustness of phylogenetic trees. For example, previous studies have reported negative effect on Hymenoptera [38] and Dictyoptera [80], neutral effect on Orthoptera [77] and Psocodea [37], and positive effect on Diptera [76], indicating the necessity of assessing the performance of inclusion or exclusion of third codon positions during each phylogenetic inference.

## Conclusions

Our study based on mitochondrial genomic analyses brought new insight into the high-level phylogenetic relationship within Collembola. The gene rearrangements along mitogenomes were identical within Onychiuridae and similar within Sminthuridae, suggesting that gene orders could provide useful information for inferring relationships among lineages, although their exact phylogenetic signals still need to be assessed. As expected, phylogenetic analyses based on different datasets and models yielded inequivalent tree topologies and nodal supports. In this study, the performance of analyses was increased by sequence partitioning, exclusion of third codon positions and inclusion of two rRNA genes, but not by translating nucleotides into codon or amino acid sequences or application of site- and branch-heterogeneous models. Regarding the phylogeny of Collembola, on infra-ordinal level, most families and superfamilies were strongly supported, except for Paronellidae and Hypogastruridae. Subfamilial-level relationship within Onychiuridae and Tomoceridae also required further clarification. At ordinal level, Symphypleona *s*. *s*. was most strongly supported, followed by Poduromorpha. Although Entomobryomorpha was not well supported, the recovered topology indicated future denser taxon sampling, data refining and analytical method optimization may enhance the support for this group. The relationship between elongated and spherical groups still remained a major unsolved problem. Although most analyses placed Neelipleona basal to all other orders, the possibility of artifact could not be eliminated given the relatively long branch lengths. In our results Symphypleona *s*. *s*. could be clustered with any other orders, while the highest supports were found with Entomobryomorpha. It is expected that inclusion of intermediate forms, such as Mackenziellidae and Coenaletidae, will promote the resolution of this phylogenetic problem.

## Supporting information

S1 Table Taxa under study, detail of GenBank accession numbers and sampling location. (DOCX)

S2 Table Sequence condition and gene orders of mitogenomes among studied species. (XLSX)

S3 Table Results of tree topology tests. (XLSX)

S1 Fig Phylogenetic trees obtained from 10 analyses. (PDF)
