# Supplementary material for: Phylomitogenomic analyses on collembolan higher taxa with enhanced taxon sampling and discussion on method selection
Source: PLoS One. 2020 Apr 13;15(4):e0230827. doi: 10.1371/journal.pone.0230827 (PMC7153868; doi:10.1371/journal.pone.0230827)
Supplement: S1 Table — (DOCX) [file pone.0230827.s001.docx]

**S1 Table. Taxa under study, detail of GenBank accession numbers and sampling location.**

| GenBank nos. | Family | Species | Size (bp) | Sampling Location | References |
| --- | --- | --- | --- | --- | --- |
| MK014212 | Entomobryidae | *Sinella curviseta* Brook, 1882 | 14,840 | China, Nanjing, Purple Mountain | Zhang et al. (2019) |
| MK431895 | Entomobryidae | *Dicranocentrus wangi* Ma & Chen, 2007 | 14,883 | China, Zhejiang province | This study |
| MK431900 | Entomobryidae | *Lepidocyrtus fimetarius* Gisin, 1964 | 14,698 | China, Zhejiang province, Pinghu city | This study |
| KT985987 | Entomobryidae | *Orchesella cincta* Linnæus, 1758 | 15,728 | The Netherlands | Direct Submission |
| EU016195 | Entomobryidae | *Orchesella villosa* von Linné, 1767 | 14,924 | Italy | Carapelli et al. (2007) |
| MK431896 | Paronellidae | *Cyphoderus albinus* Nicolet, 1842 | 14,836 | France | This study |
| MK409685 | Paronellidae | *Salina celebensis* (Schäffer, 1898) | 14,788 | China, Zhejiang province, Yongjia county | This study |
| NC_010533 | Isotomidae | *Cryptopygus antarcticus* Willem, 1901 | 15,297 | Antarctic Peninsula, Killingbeck Is. | Francesco et al. (2008) |
| KX863671 | Isotomidae | *Cryptopygus terranovus* (Wise, 1967) | 15,352 | Victoria Land, Emerging Is. | Direct Submission |
| KU198392 | Isotomidae | *Folsomia candida* Willem, 1902 | 15,147 | The Netherlands | Direct Submission |
| NC_024155 | Isotomidae | *Folsomotoma octooculata* (Willem, 1901) | 15,338 | Antarctic Peninsula, Devils Point – Livingston Is. | Direct Submission |
| MK423967 | Isotomidae | *Paranurophorus simplex* Denis, 1929 | 9,518 | China, Nanjing, Purple Mountain | This study |
| MK431894 | Oncopoduridae | *Oncopodura yosiiana* Szeptycki, 1977 | 14,808 | China, Nanjing, Purple Mountain | This study |
| MK431898 | Tomoceridae | *Novacerus tasmanicus* (Womersley, 1937) | 15,518 | Australia | This study |
| MK423966 | Tomoceridae | *Tomocerus qinae* Yu, Yao & Hu, 2016 | 15,045 | China, Nanjing, Purple Mountain | This study |
| MK409686 | Hypogastruridae | *Ceratophysella communis* （Folsom, 1898） | 15,331 | China, Henan province, Zhumadian city | This study |
| AY191995 | Hypogastruridae | *Gomphiocephalus hodgsoni* Carpenter, 1908 | 15,075 | Victoria Land, Antarctica | Nardi et al. (2003) |
| EU084034 | Neanuridae | *Bilobella aurantiaca* (Caroli, 1912) | 16,312 | Italy | Direct Submission |
| EU124719 | Neanuridae | *Friesea grisea* (Schäffer, 1891) | 15,442 | Victoria Land, Kay Is. | Torricelli et al. (2010) |
| NC_006074 | Onychiuridae | *Thalassaphorura orientalis* Stach, 1964 | 12,984 | Shanghai Botanical Garden | Cook et al. (2005) |
| MK423968 | Onychiuridae | *Thalassaphorura encarpata* (Denis, 1931) | 15,213 | China, Jilin province, Gongzhuling City | This study |
| NC_002735 | Onychiuridae | *Tetrodontophora bielanensis* (Waga, 1842) | 15,455 | Passo San Boldo, Italy | Nardi et al. (2001) |
| MK431897 | Tullbergiidae | *Mesaphorura yosii* (Rusek, 1967) | 14,833 | China, Shanghai | This study |
| NC_006075 | Poduridae | *Podura aquatica* Linnæus, 1758 | 13,809 | China, Shanghai Botanical Garden | Cook et al. (2005) |
| MK431893 | Neelidae | *Neelides* sp. | 13,858 | China, Zhejiang province, Lishui city | This study |
| MK423965 | Dicyrtomidae | *Ptenothrix huangshanensis* Chen & Christiansen, 1996 | 15,152 | China, Anhui province, Huangshan city | This study |
| MK423969 | Katiannidae | *Sminthurinus signatus* (Krausbauer, 1898) | 5,459 | France | This study |
| MK423964 | Sminthurididae | *Sminthurides bifidus* Mills, 1934 | 14,161 | China, Nanjing, Purple Mountain | This study |
| KY618680 | Bourletiellidae | *Bourletiella arvalis* (Fitch, 1862) | 14,794 | Monsindoli, Siena, Italy | Direct Submission |
| NC_010536 | Sminthuridae | *Sminthurus viridis* (Linnæus, 1758) | 14,817 | Siena, Italy | Direct Submission |
| MK431899 | Sminthuridae | *Lipothrix lubbocki* (Tullberg, 1872) | 15,141 | France | This study |
