# Supplementary figures and images for: Phylomitogenomic analyses on collembolan higher taxa with enhanced taxon sampling and discussion on method selection
Source: PLoS One. 2020 Apr 13;15(4):e0230827. doi: 10.1371/journal.pone.0230827 (PMC7153868; doi:10.1371/journal.pone.0230827)

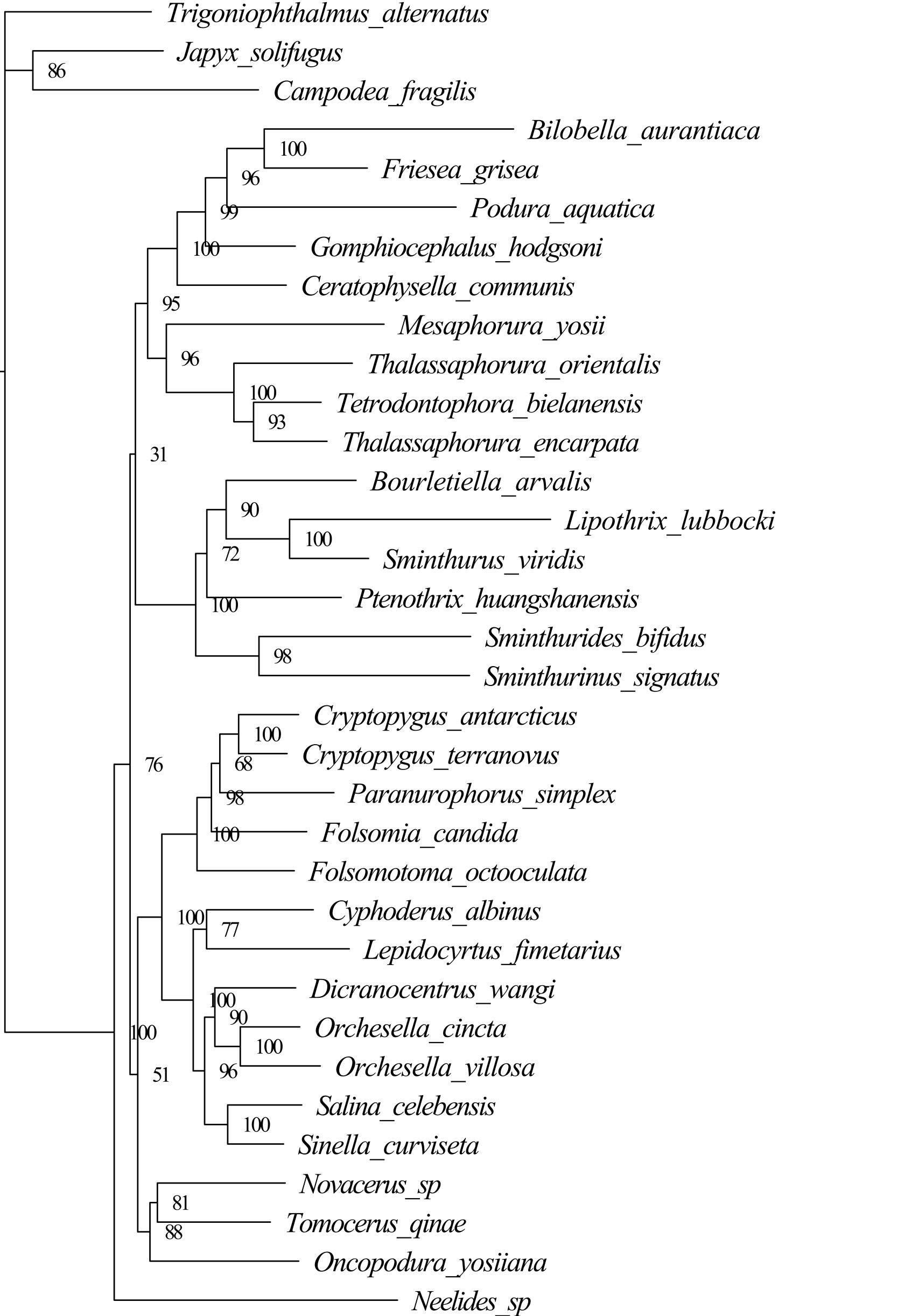

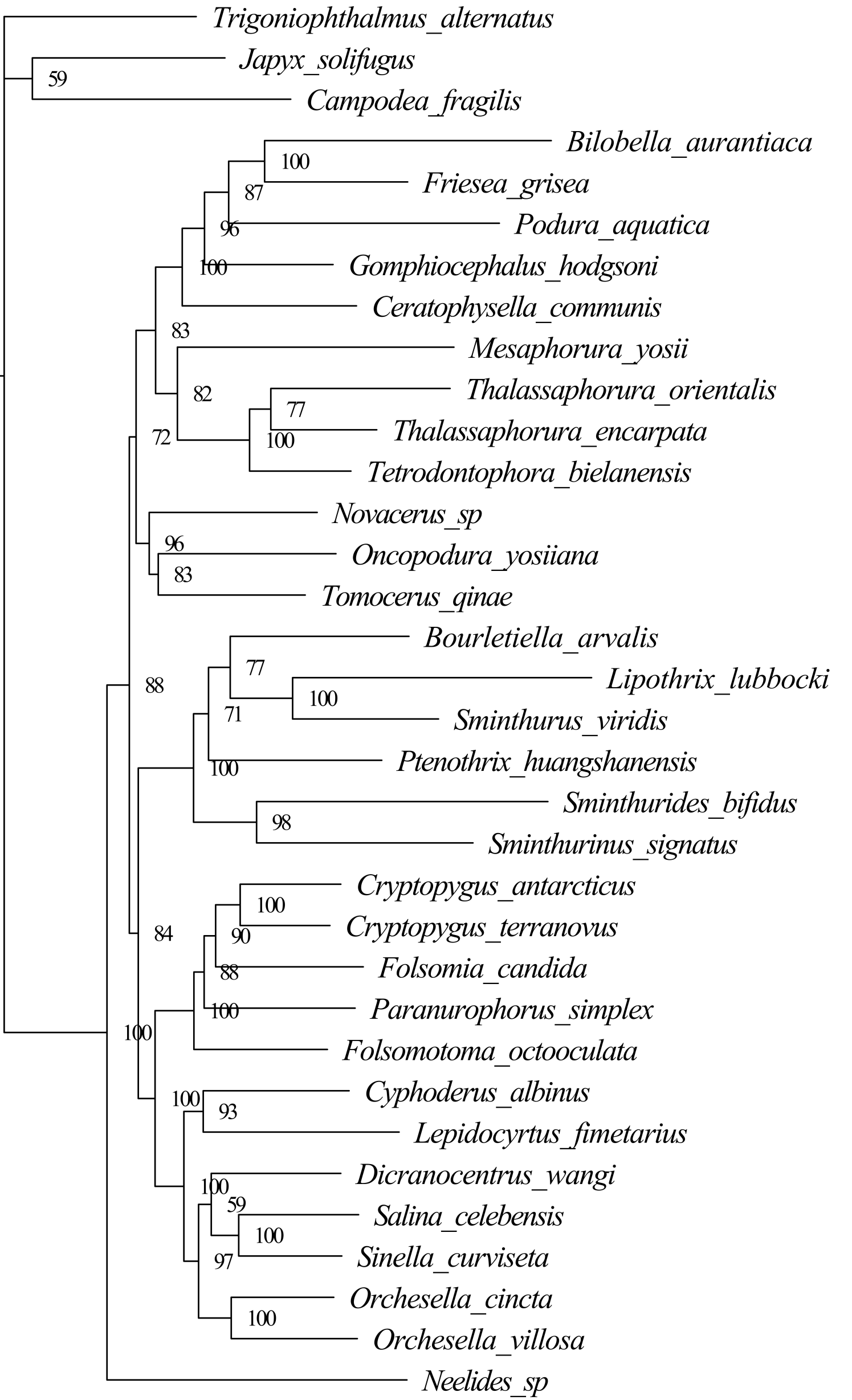

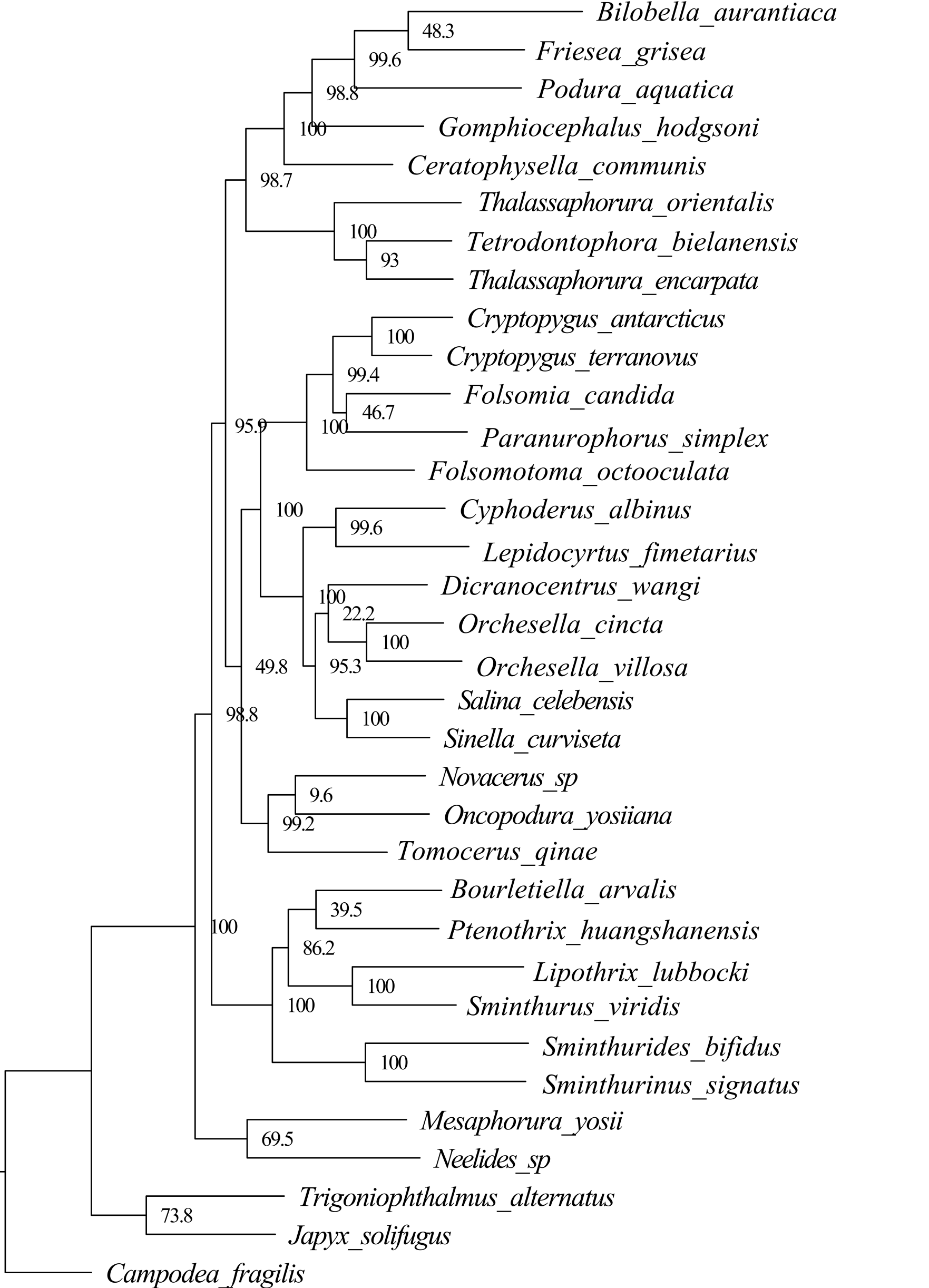

3.0

13fna\_CODON5\_GHOST

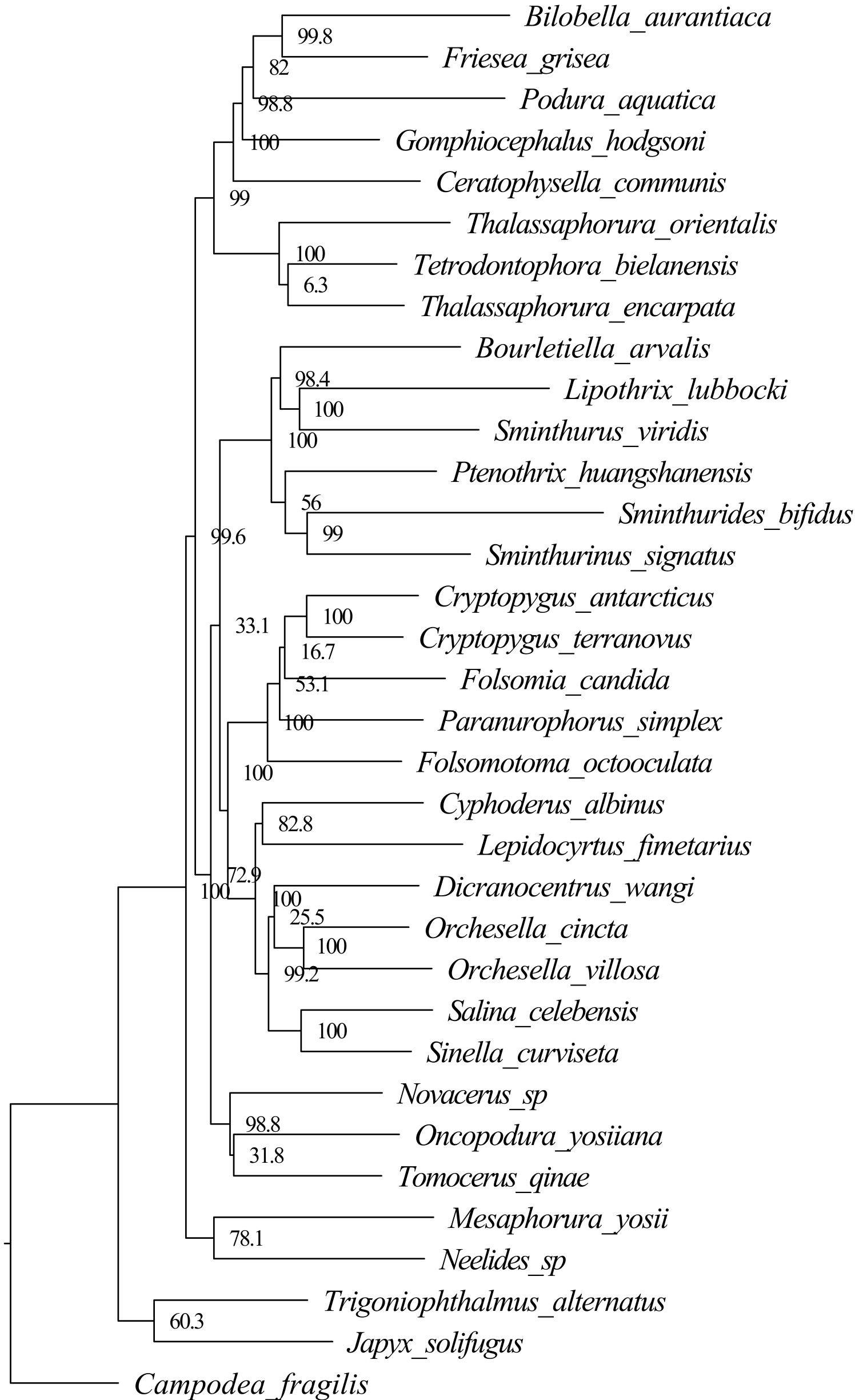

0.2

13fna\_GHOST

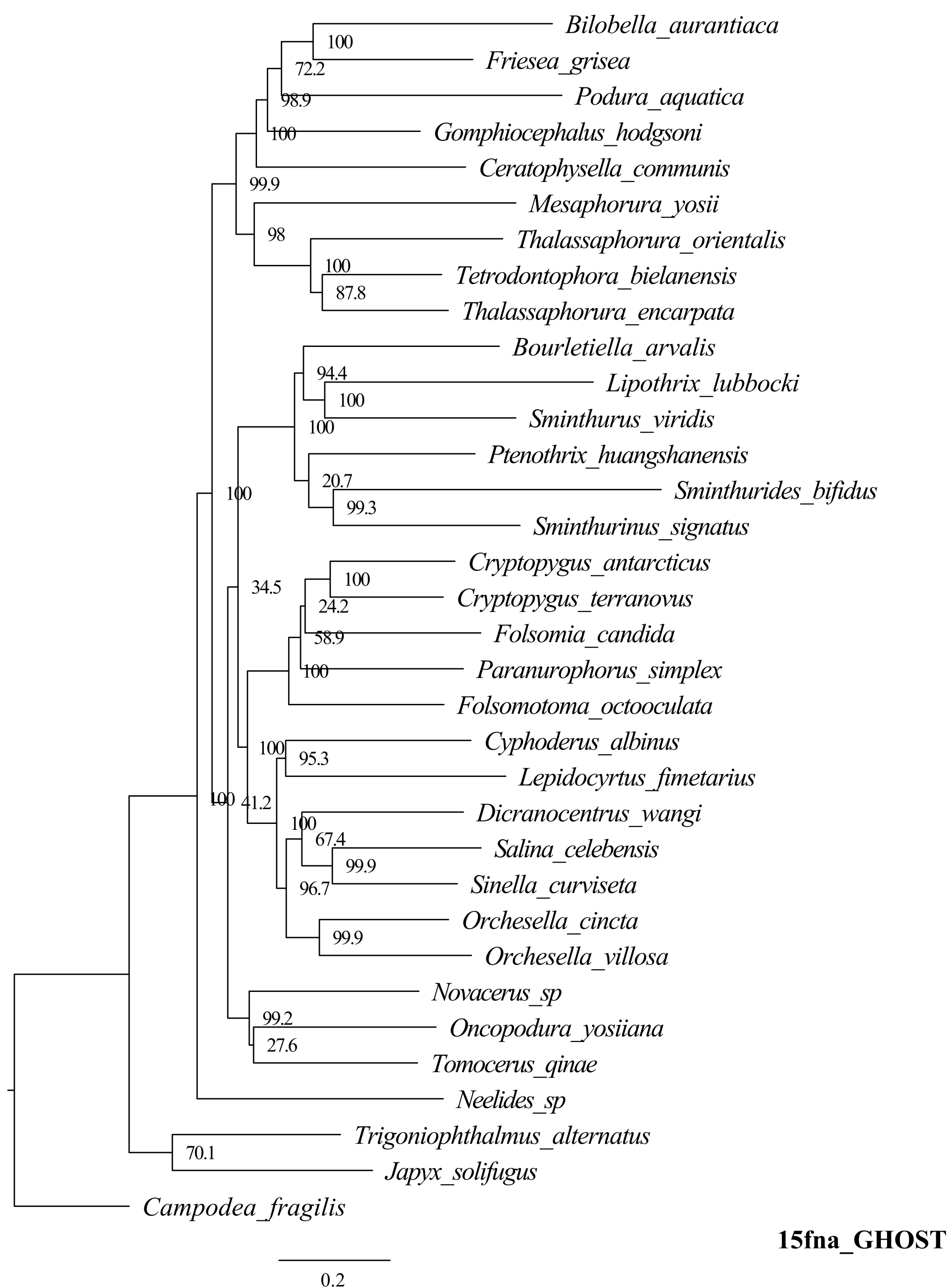

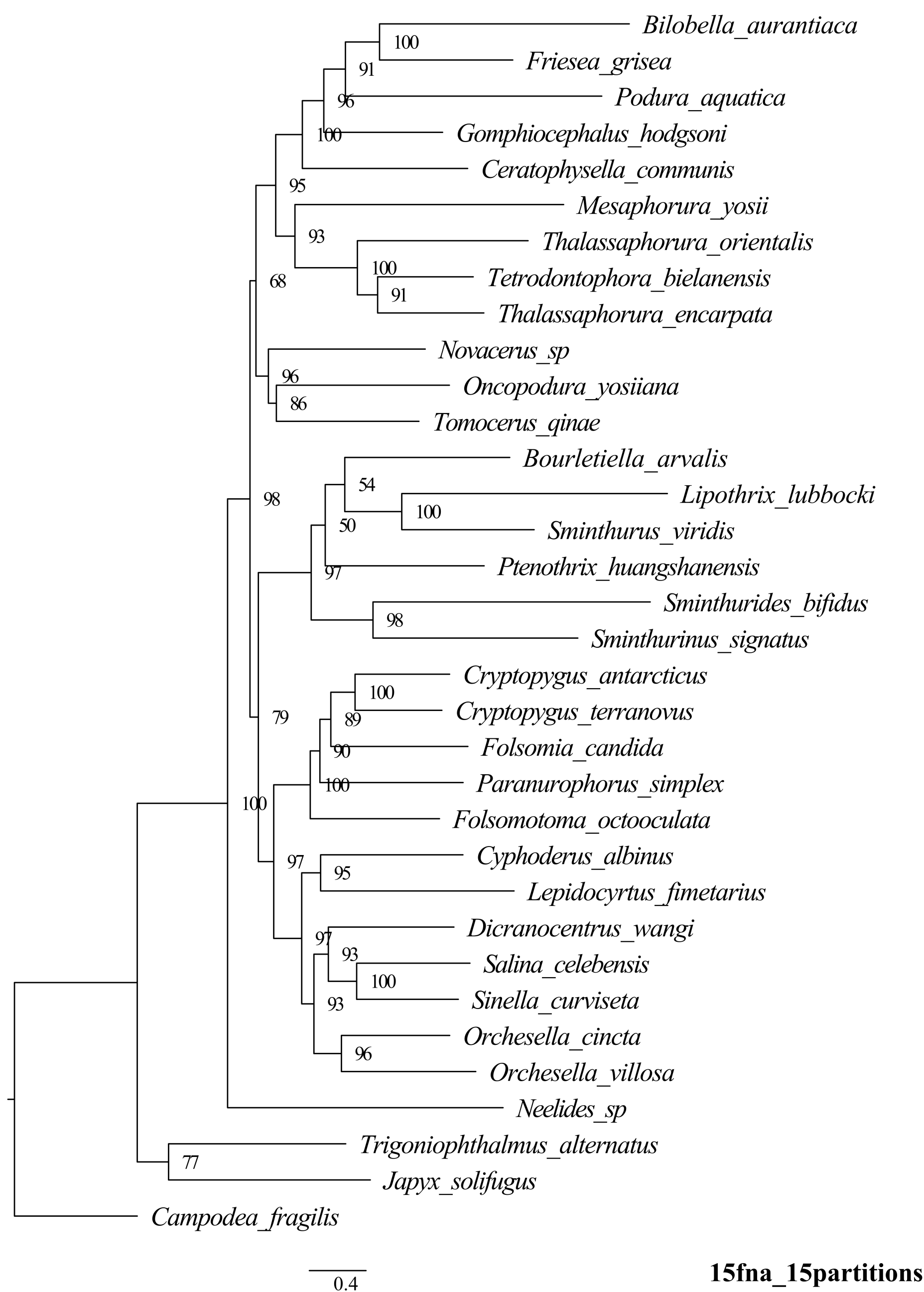

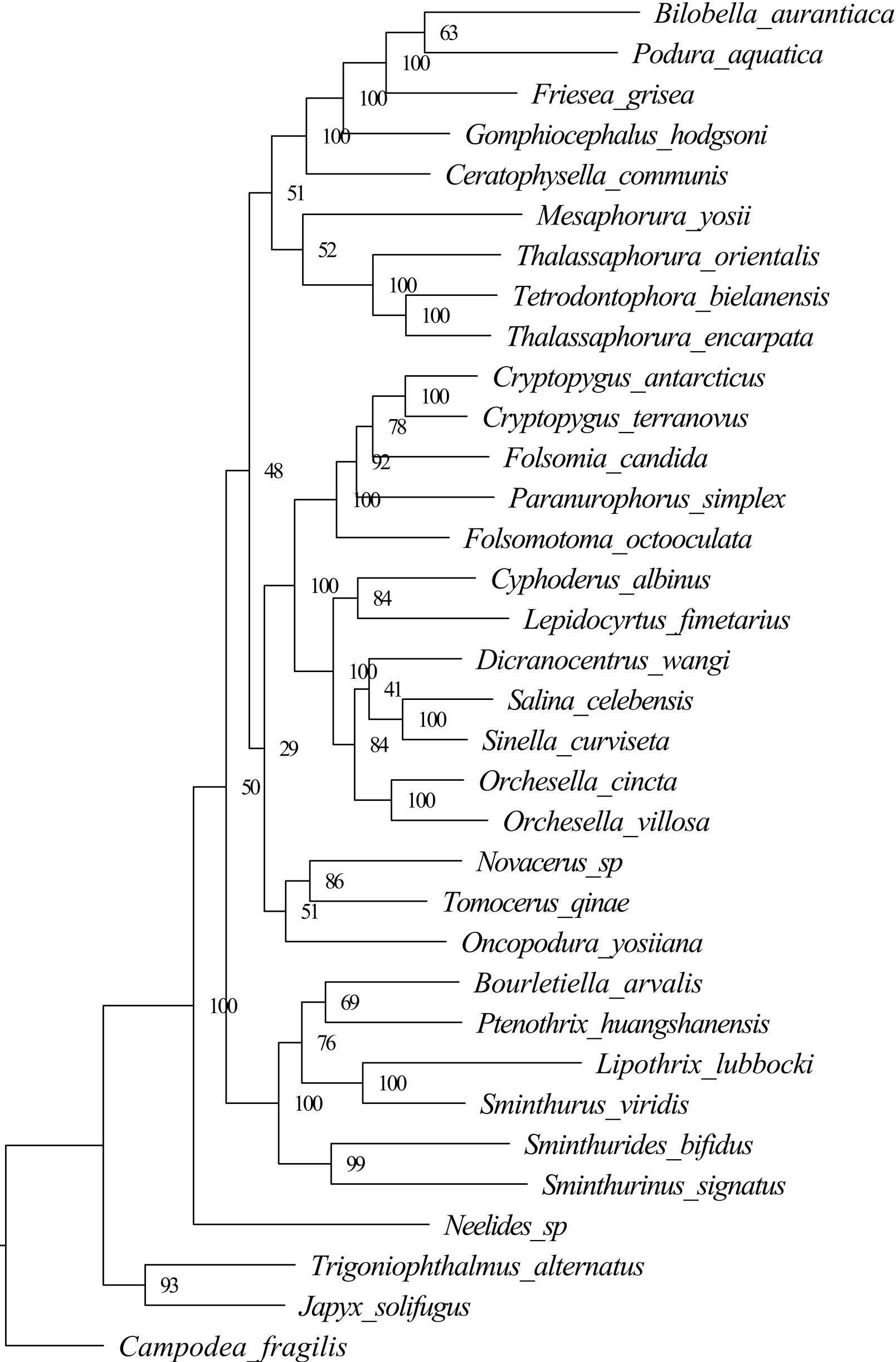

0.1

13faa\_13partitions

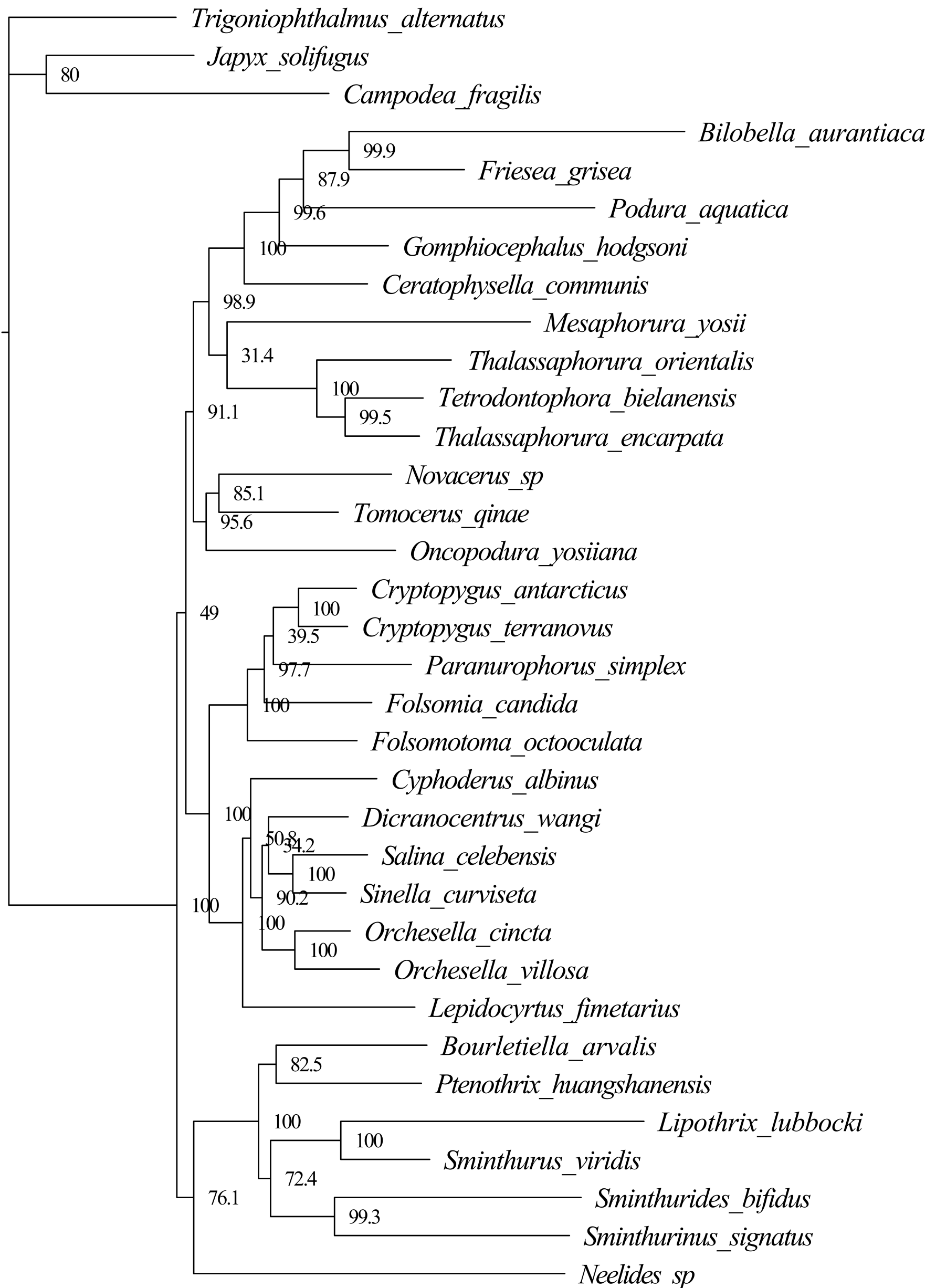

0.3

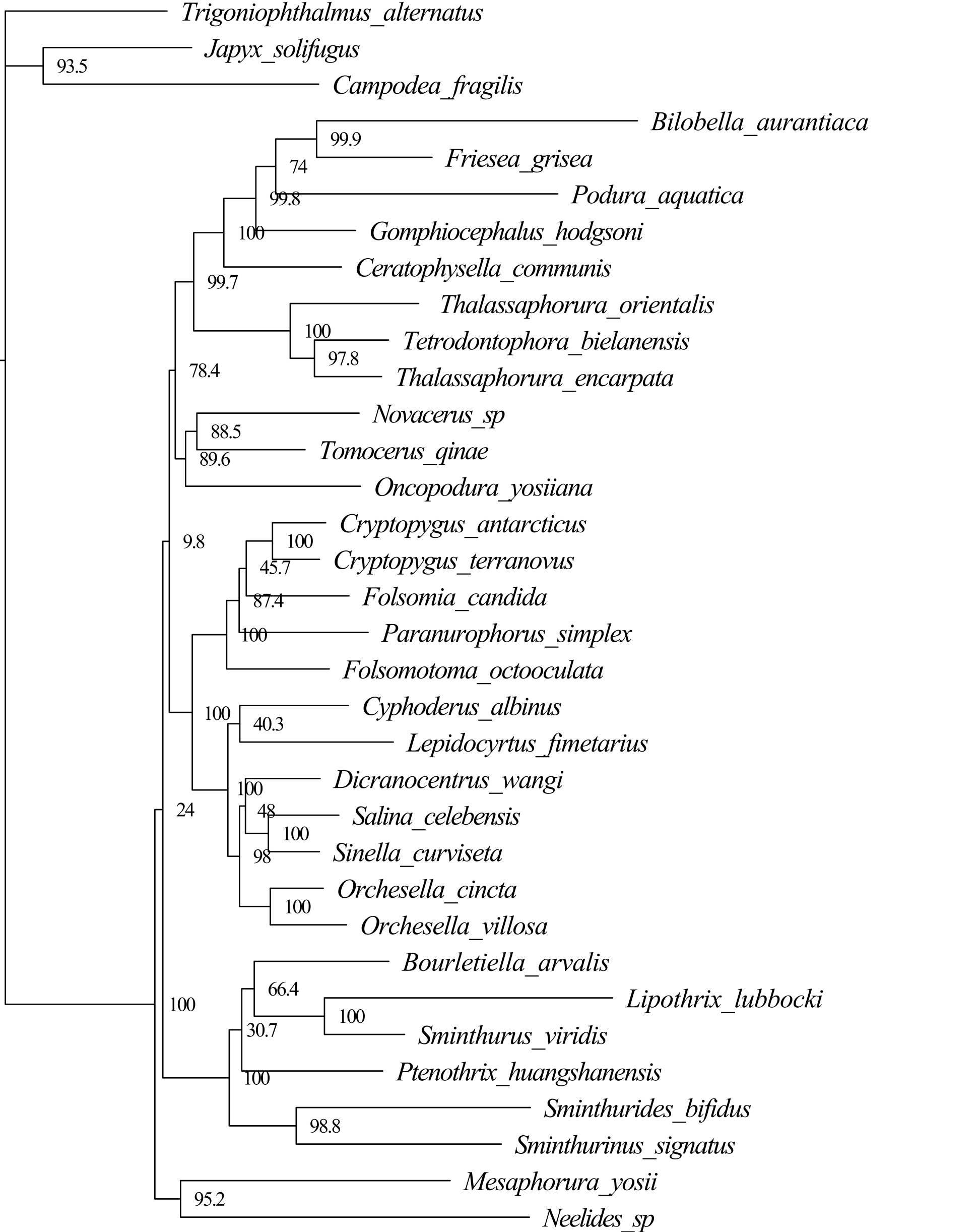

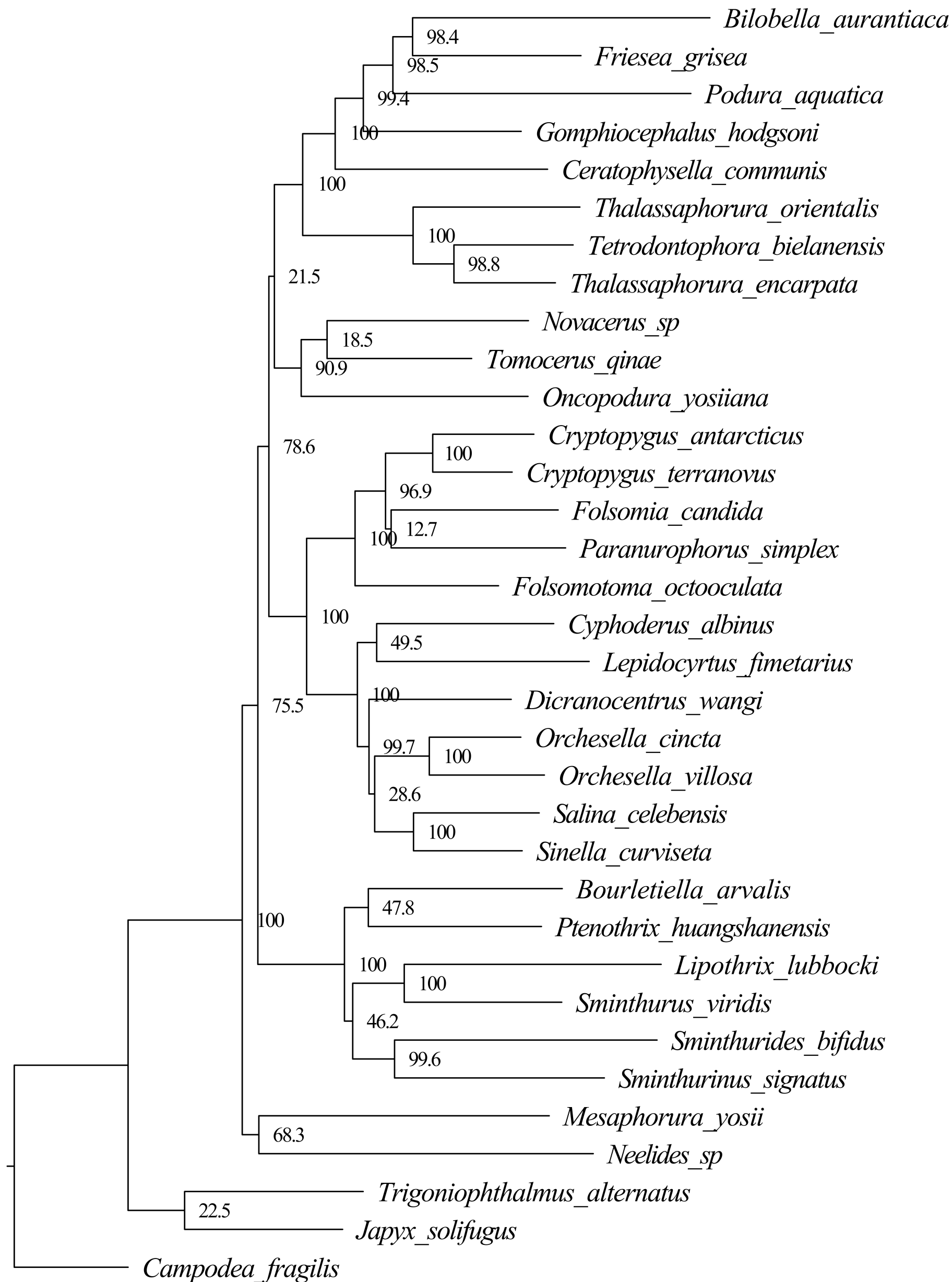

13faa\_unpartition\_GHOST\_LG

0.2

Supplement: S1 Fig — (PDF) [file pone.0230827.s004.pdf]
